# MicroRNA Biomarkers in IBD—Differential Diagnosis and Prediction of Colitis-Associated Cancer

**DOI:** 10.3390/ijms21217893

**Published:** 2020-10-24

**Authors:** Jaslin P. James, Lene Buhl Riis, Mikkel Malham, Estrid Høgdall, Ebbe Langholz, Boye S Nielsen

**Affiliations:** 1 Department of Pathology, Herlev University Hospital, 2730 Herlev, Denmark; Lene.Buhl.Riis@regionh.dk (L.B.R.); Estrid.Hoegdall@regionh.dk (E.H.); 2 The Pediatric Department, Copenhagen University Hospital, 2650 Hvidovre, Denmark; Mikkel.malham.knudsen.01@regionh.dk; 3 The Pediatric Department, Holbæk Sygehus, 4300 Holbæk, Denmark; 4 Gastroenheden D, Herlev University Hospital, 2730 Herlev, Denmark; Ebbe.Langholz@regionh.dk; 5 Institute for Clinical Medicine, University of Copenhagen, 2200 Copenhagen, Denmark; 6 Bioneer A/S, Hørsholm, Kogle Allé 2, 2970 Hørsholm, Denmark; BSN@bioneer.dk

**Keywords:** biomarkers, circulating miRNA, colitis-associated cancer (CAC), Crohn’s disease (CD), inflammatory bowel disease (IBD), microRNA (miRNA), ulcerative colitis (UC)

## Abstract

Inflammatory bowel disease (IBD) includes Crohn’s disease (CD) and ulcerative colitis (UC). These are chronic autoimmune diseases of unknown etiology affecting the gastrointestinal tract. The IBD population includes a heterogeneous group of patients with varying disease courses requiring personalized treatment protocols. The complexity of the disease often delays the diagnosis and the initiation of appropriate treatments. In a subset of patients, IBD leads to colitis-associated cancer (CAC). MicroRNAs are single-stranded regulatory noncoding RNAs of 18 to 22 nucleotides with putative roles in the pathogenesis of IBD and colorectal cancer. They have been explored as biomarkers and therapeutic targets. Both tissue-derived and circulating microRNAs have emerged as promising biomarkers in the differential diagnosis and in the prognosis of disease severity of IBD as well as predictive biomarkers in drug resistance. In addition, knowledge of the cellular localization of differentially expressed microRNAs is a prerequisite for deciphering the biological role of these important epigenetic regulators and the cellular localization may even contribute to an alternative repertoire of biomarkers. In this review, we discuss findings based on RT-qPCR, microarray profiling, next generation sequencing and in situ hybridization of microRNA biomarkers identified in the circulation and in tissue biopsies.

## 1. Introduction

Inflammatory bowel disease (IBD) refers to Crohn’s disease (CD) and ulcerative colitis (UC). In UC, inflammation generally includes the rectum and extends towards the coecum and remains confined to the colon. In contrast, in CD, inflammation can involve any part of the gastrointestinal tract (GI) from the oral cavity to the anus. Both CD and UC are associated with multiple pathogenic factors such as environmental changes, the array of susceptibility gene variants, qualitatively and quantitatively abnormal gut microbiota and broadly dysregulated immune response [1]. Although CD and UC have some common pathological and clinical characteristics, they have several different attributes that imply that they are two distinct disease subtypes. In CD, fissuring ulceration and sub-mucosal fibrosis can be observed along with granulomatous inflammation. In UC, the inflammatory process always involves the rectum [2] and general histological findings include crypt distortion, infiltration of lymphocytes and granulocytes and chronic inflammation, usually confined to the lamina propria [3]. The diagnosis of IBD is usually established by a collective assessment of clinical presentation and endoscopic, histopathological, radiographic and laboratory findings. A definitive diagnosis of IBD cannot be made without detailed endoscopic and histologic assessment [4]. However, a subset of IBD cases cannot be classified as either CD or UC but are categorized as IBD unclassified (IBDU). Molecular biomarkers may support differential diagnosis of IBDU cases into CD or UC, or even be helpful in determining if IBDU represents a unique IBD diagnostic entity.

IBD starts developing at a younger age, including in infants [5], and is often characterized by a considerable diagnostic and therapeutic challenge because of the disease’s clinical features and associated complications. The prevalence of IBD in the Western world is projected to be up to 0.5% of the overall population [6]. In Denmark, where one of the highest annual incidence rates of IBD in Europe is seen, the incidence has been increasing over the last three decades [7]. In 2013, the incidence was 9.1 per 100,000 persons and 18.6 per 100,000 persons for CD and UC, respectively [8]. Since the turn of the 21st century, IBD has become a global disease with accelerating incidence rates also in developing countries whose societies have adopted a western diet and lifestyle. Although the incidence rate has become steady in western countries, the burden remains high, as prevalence exceeds 0.3%. The chronical inflammatory condition in the affected colon of IBD patients has been linked to development of neoplastic lesions in the colon. Several studies have shown a higher incidence of colorectal cancer (CRC) in IBD patients [9,10,11]. No biomarkers exist for the identification of IBD patients at risk of developing colitis-associated cancer (CAC), strongly advocating for more translational research in this field.

In this review, we give an overview of microRNAs (miRNAs) as candidate biomarkers in the IBD diagnostic assessment. Changes in miRNA levels are associated with disease development and can be measured both within the diseased tissue and in the circulation by a variety of molecular methods. MiRNAs have been found to be well conserved in archived tissue specimens, enabling retrospective analyses of clinical sample cohorts.

## 2. MicroRNA—An Introduction

MiRNAs play a central role in the regulation of several immune-mediated disorders including IBD [12,13,14]. MiRNAs are a group of small noncoding RNAs, approximately 18–22 nucleotides [15] which are found conserved across species. Their discovery was first described first in 1993 in Caenorhabditis elegans [16]. MiRNAs are transcribed as primary transcripts by RNA polymerase, processed into a precursor miRNA by the RNase III enzyme, Drosha, and exported from the nucleus to the cytoplasm. The precursor miRNA is cleaved by the RNase III enzyme, Dicer, into its mature form, which becomes stably incorporated into an RNA induced silencing complex (RISC). The miRNA guides the binding of the RNA-induced silencing complex to complementary sequences in the 3′-untranslated regions (UTR) of target mRNA molecules, resulting in either mRNA degradation or translational inhibition [17]. During stages of miRNA biogenesis, several factors can influence the development of the mature miRNA. These include regulation of transcription, cleavage of the stem loop structures by Drosha and Dicer enzymes, and editing as well as regulation of miRNA turnover. Each of these mechanisms acts as part of a signaling network that modulates gene expression in response to cellular or environmental changes.

MiRNA expression has been shown to be of importance in a wide variety of human diseases such as cancer, autoimmune, cardiovascular, and neurodegenerative diseases [14,18,19,20,21,22,23,24]. The miRNAs not only circulate in the human peripheral blood in a stable form, they are also present in other body fluids such as urine, saliva, milk, cerebrospinal fluid, and feces [25,26,27,28]. The miRNAs are engaged in disease origin and development, and some are pathology-specific [29], thus, changes in miRNA expression profiles have been addressed for applications in early detection as well as prognostics, diagnostic classification and drug response prediction.

## 3. MiRNAs in IBD

In IBD, miRNAs have been found to be involved in pathogenesis and have been identified as both diagnostic biomarkers and therapeutic targets [30]. Most of the recent research in the IBD field has measured levels of circulating miRNAs in body fluids such as blood or feces, and in homogenized tissue biopsies using techniques like microarray profiling, RT-qPCR, and NGS [27,31,32,33,34]. Studies have also performed tissue miRNA expression analysis using in situ hybridization (ISH) methods [35,36,37]. ISH methods for expression analyses of miRNAs can determine the cellular origin of miRNA expression and can offer insight into the biology of the disease mechanisms involved. Local expression levels of miRNAs can greatly vary from those of circulating miRNAs, e.g., due to contribution of miRNAs from circulating cells. Esquela-Kerscher and Slack [38] proposed that tumor cells release miRNAs into the neighboring microenvironment and enter circulation during angiogenesis. Some studies suggest that this likely occurs through exosomal release from cells [39,40]. Changes in the levels of circulating miRNA may occur due to other inflammatory reactions or the host immune response rather than only due to the intrinsic changes within the lesion [41]. Thus, as discussed further below, it is not surprising that miRNAs analyzed in tissue biopsies poorly correlate with those found in the circulation [42].

There is an increasing interest in exploring epigenetic mechanisms in common diseases, with notable progress in characterizing the contribution of miRNAs [43]. In their 2008 study, Wu et al. found that miRNAs regulate colonic epithelial cell-derived chemokine expression and were the first to relate miRNAs to IBD [44]. The field of miRNA research has grown rapidly after their discovery in human disease biology including in IBD [43]. We have listed a series of IBD-related miRNA studies from recent years in Table 1, with a focus on sample type and quantitative method. MiR-21, miR-155, and miR-31 have repeatedly been identified and seem to be the most studied miRNAs related to IBD [15,19,35,45,46,47,48]. MiR-21 is possibly the most intriguing miRNA involved in IBD, with associations between miR-21 and IBD being replicated in several studies, as well as functional relevance reported in mouse models of IBD [19,23,24,30,35,49]. Each miRNA can potentially target hundreds of mRNAs resulting in mRNA destabilization and/or inhibition of translation, however, restricted to a specific cellular context, the number of relevant targetable transcripts is probably quite low.

MiRNAs regulate important cellular functions such as cell differentiation and proliferation and signal transduction and apoptosis and exhibit highly specific regulated patterns of gene expression [15]. In autoimmune diseases, miRNAs can act through interference with inflammatory signaling pathways, such as the nuclear transcription factor kappa B (NF-κB) pathway, IL23/IL23R pathway, and IL-6/STAT3 pathway [50,51,52,53,54]. Studying the RhoB pathway of cell adhesion in UC mucosa and cultured colon cancer cells, Yang et al. [36] examined the role of miR-21 in regulation of intestinal epithelial barrier function and found that miR-21 induced the degradation of RhoB mRNA, reduction in RhoB protein, causing loss of tight junctions in intestinal epithelial cells. Tian et al. showed miR-31 to be highly expressed in tissues from IBD patients, and miR-31 reduced the inflammatory response in the Dextran Sodium Sulphate (DSS)-induced colitis mouse model (see below), by preventing the expression of inflammatory cytokine receptors such as IL7R and IL17RA and signaling proteins such as GP130 [55]. Another study based on the DSS model showed that miR-155 directly binds to SHIP-1 mRNA and causes a significant decrease in SHIP-1 levels, which regulate cell membrane trafficking, and thereby contribute to the pathogenesis of colitis [56]. Taken together, these examples indicate the complexity of how miRNAs may act through signaling pathways in the biological settings of IBD.

Studies of circulating miRNAs have shown that miRNAs are potential candidates as biomarkers for diagnosing IBD and various other diseases [57,58,59,60,61]. The high stability of miRNAs in the body fluids and the ability to obtain rapid and accurate quantitative estimates are some merits of using circulating miRNAs as biomarkers in IBD [28]. MiRNAs are not only interesting tools for diagnosis, but also for potential future therapeutic applications by miRNA mimics or miRNA antagonists [62,63].

To study the pathogenesis and intricacy of IBD, the advancement of a variety of animal models has provided important information. The most extensively used mouse model of colitis utilizes DSS, a so-called chemical colitogen with anticoagulant properties, to stimulate epithelial damage. The DSS colitis model is simple and easy to administer. Acute and persistent colitis is achieved by altering the concentration of DSS and the frequency of administration [101]. A genetically engineered in vivo model that has been widely used to examine IBD etiology is the interleukin-10 (IL-10)-deficient mouse model [102]. IL-10 is an anti-inflammatory cytokine. Mutated IL-10 signaling systems shows early and aggressive expansion of systemic inflammation in IBD. IL-10 knockout mice develop spontaneous colitis and CAC [103]. Nata et al. [94] performed miRNA microarray profiling on IL-10-deficient mice and identified that several miRNAs were upregulated, including miR-146b that, through further studies, was found to contribute to increased intestinal inflammation by upregulating NF-κB. Shi et al. [95] showed that knockout of miR-21 in mice improved the survival rate in DSS-induced fatal colitis via protecting against inflammation and tissue injury. Hence, it was suggested that impaired expression of miR-21 in gut may block the onset or progression of IBD. Other animal models used in IBD research include genetically engineered mice, congenic mouse strains, chemically induced models, and cell-transfer models [104]. Most of the studies investigating miRNA expression in IBD have used high-throughput methods such as a microarray combined with RT-qPCR as a validation method for prioritized miRNAs.

## 4. MiRNA Biomarkers for IBD Diagnosis

**Figure 1:**
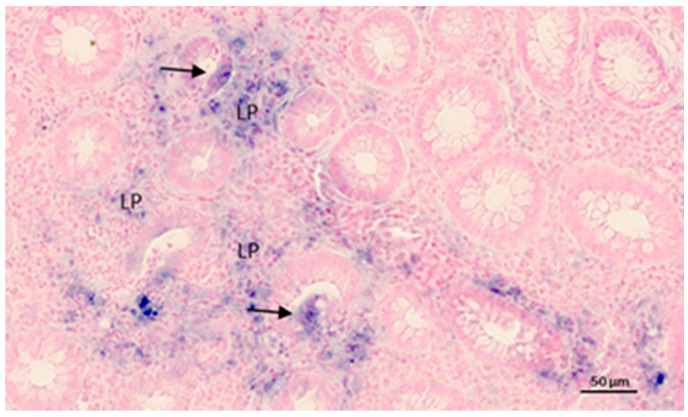
MiR-21 in situ hybridization in ulcerative colitis. The example shows the inflamed colon mucosa with transversally cut crypts and the lamina propria (indicated by LP). The miR-21 ISH signal is represented by the blue stain and is seen in inflammatory cells located in the lamina propria in and some of the epithelial cells (arrows) in some collapsed crypts. Nuclear Fast Red was used in counterstaining.

The diagnostic assessment of IBD can be challenging; particularly, discriminating CD from UC can be a diagnostic encounter in cases where the inflammatory lesions are limited to the colon. It is estimated that 10–15% of IBD cases are categorized as IBDU [105]. Although many IBDU patients are eventually reclassified as either CD or UC, approximately 75% of the IBDU cases maintain the diagnosis of IBDU, suggesting that most of the IBDU patients have a distinct diagnostic entity of a true overlap phenotype between large bowel CD and typical UC [106,107]. Recently, a study that examined colon biopsies from patients with IBD suggested miR-19a, miR-21, miR-31, miR-146a and miR-375 as a biomarker profile for discriminating CD and UC [108]. A Study by Peck et al. used a next-generation sequencing–based approach and found that a combination of miR-31-5p, miR-215, miR-223-3p, miR-196b-5p and miR-203 could stratify patients with CD according to disease behavior independent of the effect of inflammation [109]. The lack of reproducibility in miRNA profiling analyses of IBD samples in independent studies could be due to the technology applied, as well as the variation in control groups, disease activity and data normalization. It was recently reported that miR-21 is a potential diagnostic marker for discriminating CD from UC, as both RT-qPCR and quantitative ISH (qISH) identified significantly higher levels in UC compared with CD [35]. The authors suggested that miR-21 is not just an unspecific marker of inflammation, but that miR-21 is specific to the immunopathological process of UC. miR-21 ISH analyses reveal complex expression patterns, where the miR-21 staining is identified mainly in cells of the inflamed lamina propria as well as in subsets of epithelial cells of partly damaged crypt structures (example in Figure 1).

## 5. MiRNAs and CAC

Chronic inflammation is linked to the development of a variety of cancers such as CRC, pancreatic, breast, and skin cancer [110,111] and is a key hallmark of cancer [112]. Local chronic inflammation in the colon, typically caused by an unbalance in the regulation of the immune response, may damage the epithelial barrier, which induces self-sustained inflammation linked to continued microbial influx or increased levels of pro-inflammatory cytokines like tumor necrosis factor alpha (TNF) and IL-1β [110,113,114]. Increased levels of oxidants in inflamed tissue cause cell death, or more deliberately, mutations in epithelial cells that, in turn, can initiate neoplastic growth. The persisting inflammation develops into and probably shapes the tumor microenvironment that is inherent to most solid tumors with their additional presence of blood vessels and fibroblastic cells.

Patients with extensive IBD or diagnosed with IBD in childhood [115], have a shorter life expectancy that may be related to the higher risk of CRC [116,117,118]. CAC represents a type of CRC in which the IBD paved the way for the cancer, probably through mutations in K-RAS and the adenomatous polyposis coli (APC) gene [119]. The risk of CAC may be further increased in untreated IBD patients [120]. Mucosal mapping studies indicate that the chronically inflamed colonic mucosa of patients with IBD undergoes a “field change” in cancer-associated molecular alterations before there is histologic evidence of epithelial dysplasia [121,122], which is one the initial morphological changes in the stepwise progression to CRC [123,124,125].

MiRNAs are believed to take part in the inflammation in IBD and to be implicated in the process from inflammation to CRC [126]. Despite the fact that CD and UC can affect the entire colon, Ranjha et al. [127] found that CRC in UC patients developed primarily in the rectosigmoid areas of the colon, whereas other parts, such as the ascending colon, showed less frequent development of CRC. Analyzing tissue from rectosigmoid and ascending colon, the authors found differences in the miRNA expression patterns, and suggested that the local miRNA profile could contribute to the development of CRC.

MiRNAs likely play both oncogenic and tumor-suppressive roles in the carcinogenesis and progression of CRC by regulating the expression of numerous cancer-related genes. The role of the inflammatory burden has also been studied in animal models and indicates that both the initiation and the progression of colonic neoplasia can be aggravated or accelerated by the inflammatory conditions [30,126,128,129,130]. The DSS-induced colitis model has been used to study the role of multiple miRNAs in IBD and CAC, including miR-21, miR-155, and miR-301a, which will be addressed briefly in the following.

MiR-21 is one of the most prevalent miRNAs in CRC and other cancer types [131,132], and the increased expression levels in CRC are associated with poor prognosis [24,133]. MiR-21 acts on tumor-suppressor genes, like PTEN and PDCD4 [134,135,136], and is thus categorized as an oncomiR [137]. Since miR-21 is upregulated in IBD [35,44,47], and miR-21 reduction in the DSS model lowers inflammation in DSS-induced colitis [95], it is tempting to speculate that miR-21 is a key facilitator of CAC. In support of this hypothesis, a study of human IBD, found that the tumor-suppressor-programmed cell death 4 (PDCD4) was downregulated, while miR-21 was upregulated [134]. Suppression of PDCD4 and NF-κB activation was found along with reduced levels of pro-inflammatory TNF [134,135]. In addition, epithelial miR-21 upregulation in UC was reported to increase intestinal permeability, which is believed to be a key pathophysiological step in the development of IBD [138].

MiR-155 is upregulated in both UC and CD patients compared to healthy controls [48,139,140,141] and is upregulated in both tissue and blood from CRC patients, and is furthermore an indicator of poor prognosis [142,143]. MiR-155 promotes intestinal inflammation in UC and CD, probably via a variety of inflammation-related pathways [46,56,73,79,81,139]. In a recent study by Liu et al. [144,145], it was shown that miR-155 mediates intestinal barrier dysfunction in DSS-induced mice colitis through targeting the HIF-1α/TFF-3 axis. Paraskevi et al. [140] found that miR-155 is the most highly expressed UC-associated miRNA in blood samples, however, in the study by Schönauen [47], the authors did not find increased miR-155 levels in the blood from IBD patients, suggesting that more studies are needed to determine whether miR-155 is a putative blood-related biomarker.

MiR-301a is upregulated in both blood and tissue from IBD and CRC patients [76,141,146]. He et al. [76,129] found increased levels of miR-301a in peripheral blood monocytes and in the mucosa from IBD patients and in mice after administration of DSS. Using the DSS-induced IBD model in mice with an inactivated miR-301a, miR-301a was found to reduce the inflammation through the suppression of BTG anti-proliferation factor 1 (BTG1) and to reduce the development of CAC [129]. Thus, miR-301a should be investigated in future studies to establish possible use as a clinically relevant diagnostic biomarker in IBD and for prediction of CAC.

## 6. MiRNAs as Predictive Biomarkers and in IBD Treatment

The goal of the treatment of IBD patients is to obtain remission and mucosal healing, and thereby lower surgery rates. The classical therapies include corticosteroids, thiopurines, and amino salicylates (5-ASA), which have been in use for decades. 5-ASA has minor side effects [147,148] and it is very effective for treating mild to moderate UC patients, but not recommended for treatment of patients with CD [149]. The last-line medical treatment in IBD is administration of biologics targeting key elements in the inflammatory process. Anti-TNF therapies include TNF inhibitors that antagonize the pro-inflammatory cytokine TNF [150]. The use of anti-TNF therapy has improved long-term outcomes for IBD patients [149,151]. Even though TNF inhibitors have improved the overall conditions for a large group of IBD patients, approximately 30% of patients fail to respond to TNF inhibitors (primary non-responders), and up to 50% of the patients who initially benefited from treatment with TNF inhibitors lose the response over time (secondary non-responders) [152,153]. Thus, identifying predictors of responders/non-responders and choosing a treatment strategy according to biomarker profiles could improve overall IBD disease management. Interestingly, Morilla et al. [154], found that nine miRNAs, together with five clinical factors correlated with response to treatment of IBD patients, and that neural-network-developed algorithms based on certain miRNA levels identified responders to the anti-TNF antibody therapy, infliximab, vs. non-responders.

Currently used therapies in IBD also include Ustekinumab, Vedolizumab and Tofacitinib. Ustekinumab is a monoclonal antibody against IL-12 and IL-23, which is used in patients with moderate to severe CD who are resistant to anti-TNF treatment [155]. Considering the efficacy of ustekinumab, it is possible to extrapolate the efficacy of miR-29 mimicry as a mechanism to reduce IL-23 levels [12]. With respect to potential secondary target effects, miR-29c has been described as a tumor-suppressor in liver cancer [156]. Vedolizumab binds specifically to α4β7-integrin on T-helper lymphocytes. Blocking the α4β7-integrin results in anti-inflammatory activity that is caused by the inhibition of leukocyte adhesion to endothelial cells, which consequently reduces leukocyte recruitment to affected tissue [157]. Previous studies have suggested a similar effect of miRNAs in the posttranscriptional regulation of leukocyte trafficking [158]. Harris et al. [158] described how endogenous miR-126 inhibits leukocyte adherence through the regulation of an intercellular adhesion molecule expressed by endothelial cells (VCAM-1). Tofacitinib is a janus kinase (JAK) inhibitor, approved for treating moderate-to-severely active UC patients who have deteriorated disease and did not improve after conventional or antibody-based therapies [159,160]. Pathak et al. [51] identified SOCS1, a potent molecular switch that tunes the JAK pathway that is also a direct target of miR-155.

In general, miRNA-based therapies comprise two fundamental strategies: miRNA antagonism and mimicry [161,162,163,164]. Physiologic miRNA over-expression resulting in pathologically reduced target gene expression can be hindered by using miRNA antagonists, while reduced miRNA expression resulting in enhanced target function can be restored by utilizing miRNA mimics [12]. A study by Lu et al. [56] reported that a so-called antagomir towards miR-155 alleviated DSS-induced intestinal inflammation in mice, and the authors propose that anti-miR-155 could be a promising candidate for a novel IBD therapy. Jin et al. conducted a study on miR-133a and its target UCP2 (mitochondrial uncoupling protein 2) using the DSS-induced IBD mouse model [77]. miR-133a levels were found to be decreased upon DSS treatment, and by introducing a miR-133a mimic, the DSS-induced IBD was alleviated, suggesting that miRNA mimics could also function as therapy in IBD [77].

## 7. Circulating miRNAs vs. Tissue miRNAs

It is of importance to determine whether miRNA dysregulation in the circulation reflects similar changes in the lesion. The detection and quantification of circulating miRNAs and the interpretation of their impending role as novel non-invasive biomarkers could be very beneficial in the diagnosis and treatment of IBD. As mentioned above, miRNAs can be detected in distinct body fluids such as saliva, plasma or urine [32]. Current diagnostic and predictive findings in IBD on miRNA expression profiling have mainly focused on the assessment of miRNAs in blood. Even though blood samples can be relatively easily obtained from IBD patients, miRNA measurement in blood samples, as with other biological samples, comes with some inherent obstacles, such as sample procurement, storage, measurement platform and normalization of the acquired data. Circulating miRNAs may derive from both the diseased tissue and by leakage from the normal vascular network and circulating cells. Obtaining tissue samples, on the other hand, requires an invasive procedure, where small biopsies from the affected part of the bowel are obtained during endoscopy. The tissue samples can be either frozen or fixed in formalin and paraffin-embedded (FFPE) for histological examination. MiRNAs can be isolated from both fresh-frozen and FFPE tissue samples. Normalization of miRNA data from both blood and tissue samples is an important step for data interpretation in the comparison between patients, and between different study cohorts. MiRNA expression levels measured in tissue samples will have been derived from cells in the normal tissue and from activated cells in the lesion. To be able to find the same miRNAs in the tissue as in the circulation would require substantial expression in the lesion and/or for the background level in the circulation to be low. Thus, it may not be surprising that the study by Iborra et al. [42] of tissue biopsies and peripheral blood showed that none of the serum miRNAs corresponded with tissue miRNAs in the CD and UC patients. Feces samples represent another liquid biopsy that is relevant in relation to IBD and may be better linked to expression levels in the diseased mucosa than to the levels in the blood circulation. Schönauen et al. [47] analyzed both serum and fecal miRNAs in IBD and found increased levels of miR-16, miR-21, and miR-223 in both sera and feces from the IBD patients compared to controls. In addition, the authors found that fecal levels, but not sera levels, of miR-16 and miR-223 correlated with clinical parameters, like C-reactive protein and calprotectin. Thus, fecal samples seem to be a promising alternative to blood for miRNA profiling in IBD.

As seen in Table 1, most miRNA studies have used high-throughput methods such as RT-qPCR and microarray for miRNA analysis in IBD. It is important to note that these techniques require homogenization of the tissue to isolate the miRNAs. Homogenization of the tissue will degrade the spatial arrangement and, hence, will give an overview of the miRNA expression at the tissue level. ISH using Locked Nucleic Acid (LNA) probes is a method that allows detection of miRNAs in tissue sections [165]. Detection of miRNAs at the cellular level determines the cellular origin of expression and can provide evidence on expression levels in different cell populations and tissue compartments [35]. More knowledge about the cellular localization of miRNAs in the framework of IBD is needed as this will provide a vital link between the growing amounts of miRNA biomarkers discovered in IBD and functional studies identifying various miRNA target genes. Thorlacius-Ussing et al. used quantitative ISH on IBD tissue samples and showed that miR-126 levels are increased in UC and expressed in endothelial cells and miR-21 is expressed in subsets of monocytes/macrophages and T cells [35]. As also suggested from Figure 1, ISH data provide information of contextual expression in the tissue, as exemplified by focal upregulation in certain tissue compartments. Simple histological analysis from ISH analysis if IBD tissue can often determine if a miRNA is expressed in the epithelial or stromal (lamina propria) compartment. Nielsen and Holmstrøm presented a method to combine miRNA ISH using LNA-containing probes with immunohistochemical detection of cell-specific protein markers in order to better characterize the miRNA’s cellular characteristics [166]. This approach could also be used to monitor parallel downregulation of the specific downstream target protein. MiRNA ISH is a powerful tool when also combined with parallel characterization of the cell population in question and of mRNAs using combined staining methods [23]. Thus, for better understanding of the role of miRNAs in IBD and CAC, miRNA ISH analyses will be a helpful tool both for validating expression and for deciphering the related inflammatory molecular context.

## 8. Concluding Remarks

MiRNAs in IBD research started with the extensive pioneering work by Wu et al. in 2008 [44], who found altered expression of several miRNAs in tissue from IBD patients. Since then, there have been tremendous advancements in the field both regarding mechanistic studies and studies evaluating the use of miRNAs as diagnostic and predictive biomarkers in IBD. The miRNAs are involved in the regulation of the NF-κB and the IL-6 pathways, regulating the inflammatory activity. The inflammation is fueled by cytokines like TNF, which is currently a key therapeutic target. Thus, the dysregulated miRNAs may be considered also as therapeutic targets in IBD. Tracking the immune status in IBD based on miRNA signatures determined from liquid or tissue biopsies, may be powerful for designing individualized therapies that could be, e.g., combinations of conventional drugs and biologically active drugs, like anti-TNF. In this review, we discussed the possibility of using miRNA expression profiles to understand the link between inflammation in IBD and CAC, where animal models of IBD have provided new information on the role of miRNAs both as biomarkers and as possible therapeutic targets. Future studies may apply new sequencing techniques and histology-based multiplexing analyses in well-annotated independent patient cohorts to address the possible value of miRNAs as diagnostic and predictive biomarkers.

## Figures and Tables

**Table 1:** A summary of studies on microRNA research in inflammatory bowel disease (IBD). CD: Crohn’s disease, UC: Ulcerative colitis, HC: Healthy controls, RT-qPCR: Quantitative real time polymerase chain reaction, Biopsy: colon tissue biopsy, ISH: In situ hybridization, QISH: Quantitative in-situ hybridization, PBMC: Peripheral blood mononuclear cells, DSS: Dextran sodium sulphate, AOM: Azoxymethane, TNF: Tumor necrosis factor alpha.

#	MiRNAs	Disease Subtype	Sample Type	Techniques Used	Outcome	Reference
1	miR-16, miR-29a, miR-199a-5p, miR-363-3p, miR-340, miR-532-3p, miRplus-1271, miR-140-3p, miR-127-3p, miR-196b, miR-877, miR-150	CD, UD, HC	Serum, Biopsy	RT-qPCR, Microarray	Mixed outcomes	[42]
2	miR-223-3p, miR-31-5p	CD, HC	Biopsy	Nano string	Mir-223-3p expression showed age and sex effects and miR-31-5p expression was driven by location	[45]
3	miR-29b	CD	Fibroblasts	RT-qPCR	MCL-1 is modulated in CD fibrosis by miR-29b via IL-6 and IL-8	[64]
4	miR-141, miR-200a, miR-200b, miR-200c	UC, CD	Biopsy	RT-qPCR	All investigated miRNAs were significantly down regulated in CD, and 3 of them were downregulated in UC in comparison to the normal or the least affected mucosa.	[65]
5	miR-141	UC, HC	Biopsy	Microarray, RT-qPCR	MiR-141 plays a role in the bowel inflammation of individuals with active UC via down regulation of CXCL5 expression.	[66]
6	miR-124	UC, HC	Biopsy	RT-qPCR	MiR-124 regulates the expression of STAT3. Reduced levels of miR-124 in colon tissues of children with active UC appear to increase expression and activity of STAT3.	[67]
7	miR-19b	CD, HC	Biopsy, Cell culture	RT-qPCR, ISH	MiR-19b suppresses the inflammation and prevents the pathogenesis of CD.	[68]
8	miR-590-5p	CD, HC	Human and mice tissues	RT-qPCR	Decreased miR-590-5p levels in CD.	[69]
9	miR-122	CD, HC	Biopsy	RT-qPCR, Sequencing	Significant increase of miR-122 expression in cells treated with 5′-AZA.	[70]
10	miR-10a	CD, UC, HC	Biopsy	RT-qPCR	Dendritic cell activation and Th1/Th17 cell immune responses were inhibited via miR-10a in IBD.	[71]
11	miR-192	CD, UC, HC	Biopsy	RT-qPCR, Microarray, ISH	MiR-192 with decreased expression in active UC.	[44]
12	miR-15a	CD, UC, HC	Biopsy, Cell cultures	RT-qPCR	MiR-15a negatively regulates epithelial junctions through Cdc42 in Caco-2 cells	[72]
13	miR-146a, miR-155	CD	Biopsy	RT-qPCR	MiR-146a and -155 shows increased duodenal expression in pediatric CD.	[73]
14	miR-146b-5p	CD, UC, HC	Serum	RT-qPCR	Higher expression of serum miR-146b-5p in patients with CD and UC than in HC.	[74]
15	miR-425	CD, UC, HC	Biopsy, PBMC	RT-qPCR	Increased expression of miR-425 in IBD.	[75]
16	miR-301a	IBD	PBMC, Biopsy	RT-qPCR	MiR-301a promotes intestinal mucosal inflammation via induction of IL-17a and TNF in IBD.	[76]
17	miR-125b, miR-155, miR-223 and miR-138	UC	Biopsy	RT-qPCR, Microarray	Differential expression of miR-223, miR-125b, miR-138, and miR-155 in the inflamed mucosa compared to non-inflamed mucosa and controls.	[48]
18	miR-16, miR-21, miR-155, and miR-223	CD, UC, HC	Serum, Feces	RT-qPCR	Differential expression of miR-16, miR-155, miR-21, and miR-223 in IBD.	[47]
19	miR-21	UC, HC	Biopsy	RT-qPCR, ISH	Over expression of miR-21 in UC.	[36]
20	miR-133a	IBD	Mice Tissue	RT-qPCR	MiR-133a-UCP2 pathway participates in IBD by altering downstream inflammation, oxidative stress, and markers of energy metabolism.	[77]
21	miR-20b, miR-98, miR-125b-1, let-7e	CD, UC, HC	Biopsy	RT-qPCR, Microarray	MiR-20b, miR-98, miR-125b-1, and let-7e are deregulated in patients with UC.	[78]
22	miR-155	CD, HC	PBMC	RT-qPCR, Transfection	MiR-155 regulates IL-10-producing CD24 CD27+ B Cells.	[79]
23	miR-21, miR-126	CD, UC, HC	Biopsy	RT-qPCR, qISH	Endothelial expression of miR-126 are increased in UC. MiR-21 is expressed in subsets of monocytes/macrophages and T cells.	[35]
24	miR-31	CD, UC, HC	Cell culture, Biopsy	RT-qPCR, ISH, Transfection	Expression of miR-31-3p in human colonic epithelial cells.	[80]
25	miR-21, miR-155	UC, HC	Biopsy	RT-qPCR	MiR-21 and miR-155 was highly expressed in UC.	[81]
26	miR-15	UC, HC, IBS	Biopsy	RT-qPCR	MiR-15 activates NF-κB Pathway in UC.	[82]
27	miR-143, miR-145	UC, HC	Biopsy	RT-qPCR, ISH	MiR-143 and miR-145 are down regulated in UC.	[83]
28	miR-206	UC, HC	Cell culture, Biopsy	RT-qPCR,	MiR-206 as a biomarker for response to mesalamine treatment in UC.	[84]
29	miR-193a-3p	UC, HC	Cell culture, Biopsy	RT-qPCR, ISH	MiR-193a-3p reduces intestinal inflammation in response to microbiota.	[85]
30	miR-19a	UC, HC	Biopsy, mice tissue	RT-qPCR	Reduced expression of miR-19a in human colon tissue with UC and in DSS-treated mice colitis.	[86]
31	miR-21-5p	UC, HC	Sera, rat tissue	RT-qPCR, Transfection	MiR-21-5p was down regulated in the sera and colon tissue of UC compared with healthy people and the control group.	[87]
32	miR-200b	CD, HC	Biopsy, Serum. Cell culture	RT-qPCR	MiR-200b is involved in intestinal fibrosis of CD.	[88]
33	miR-155	Colitis	Mice tissue, cell culture	RT-qPCR, Transfection	MiR-155 promotes the pathogenesis of experimental colitis by repressing SHIP-1 expression.	[57]
34	miR-31	IBD, CAC, CRC	Biopsy	RT-qPCR, Microarray, Transfection	MiR-31 expression levels as a marker for disease progression and to discriminate distinct pathological entities that co-exist in IBD.	[89]
35	miR-150	UC, HC	murine model	RT-qPCR	MiR-150 was elevated and c-Myb were down regulated in human colon with active UC compared to HC.	[90]
36	miR-122	CD	Cell culture	RT-qPCR, Transfection	MiR-122 reduces the expression of pro-inflammatory cytokines (TNF and IFN-γ) and promotes the release of anti-inflammatory cytokines (IL-4 and IL-10).	[91]
37	miR-141	CD	Murine models, Biopsy	Microarray, RT-qPCR	MiR-141 regulates colonic leukocytic trafficking by targeting CXCL12β during murine colitis and human CD.	[92]
38	miR-7	CD, HC	Cell culture, Biopsy	Transfection, RT-qPCR	MiR-7 modulates CD98 expression during intestinal epithelial cell differentiation.	[93]
39	miR-146b	IBD	IL-10 deficient mouse	Microarray, Transfection, DSS induced colitis in vivo	MiR-146b improves intestinal injury in mouse colitis.	[94]
40	miR-21	IBD	IL-10 deficient mouse, Biopsy	DSS-induced Experimental Colitis, RT-qPCR, ISH	MiR-21 is overexpressed in intestinal inflammation and tissue injury.	[95]
41	miR-215	UC, CAC	Biopsy	Nano string	MiR-215 discriminates patients who progressed to neoplasia as early as 5 years prior to the diagnosis of neoplasia	[96]
42	miR-449a	HC, CAC	DSS animal model biopsy	RT-qPCR, ISH	MiR-449a expression decreased gradually during the progression of CAC	[97]
43	miR-135a	CAC	DSS mouse model biopsy	ISH, RT-qPCR	MiR-135a in colonic cells were suppressed and up-regulating miR-135a inhibited apoptosis and inflammation of colonic epithelial cells	[98]
44	miR-146a, miR-155, miR-122	CD, UC, HC	Biopsy	RT-qPCR	Altered expression of all three miRNAs in colonic mucosa of children with IBD	[46]
45	miR-146a, miR-335, miR-26b and miR-124	CD, UC, CRC	Genome-wide expression profiles	Bioinformatics	MiR-146a, miR-335, miR-26b and miR-124 were identified in CD, UC, and CRC samples	[99]
46	miR-155	CAC, HC	AOM and DSS mouse model biopsy	Microarray, RT-qPCR	MiR-155 is upregulated in and relates to CAC	[100]
